# High Cooperativity of the SV40 Major Capsid Protein VP1 in Virus Assembly

**DOI:** 10.1371/journal.pone.0000765

**Published:** 2007-08-22

**Authors:** Santanu Mukherjee, Mahmoud Abd-El-Latif, Michal Bronstein, Orly Ben-nun-Shaul, Stanislav Kler, Ariella Oppenheim

**Affiliations:** Department of Hematology, Hadassah Medical School, Hebrew University, Jerusalem, Israel; University of Cambridge, United Kingdom

## Abstract

SV40 is a small, non enveloped DNA virus with an icosahedral capsid of 45 nm. The outer shell is composed of pentamers of the major capsid protein, VP1, linked via their flexible carboxy-terminal arms. Its morphogenesis occurs by assembly of capsomers around the viral minichromosome. However the steps leading to the formation of mature virus are poorly understood. Intermediates of the assembly reaction could not be isolated from cells infected with wt SV40. Here we have used recombinant VP1 produced in insect cells for *in vitro* assembly studies around supercoiled heterologous plasmid DNA carrying a reporter gene. This strategy yields infective nanoparticles, affording a simple quantitative transduction assay. We show that VP1 assembles under physiological conditions into uniform nanoparticles of the same shape, size and CsCl density as the wild type virus. The stoichiometry is one DNA molecule per capsid. VP1 deleted in the C-arm, which is unable to assemble but can bind DNA, was inactive indicating genuine assembly rather than non-specific DNA-binding. The reaction requires host enzymatic activities, consistent with the participation of chaperones, as recently shown. Our results demonstrate dramatic cooperativity of VP1, with a Hill coefficient of ∼6. These findings suggest that assembly may be a concerted reaction. We propose that concerted assembly is facilitated by simultaneous binding of multiple capsomers to a single DNA molecule, as we have recently reported, thus increasing their local concentration. Emerging principles of SV40 assembly may help understanding assembly of other complex systems. In addition, the SV40-based nanoparticles described here are potential gene therapy vectors that combine efficient gene delivery with safety and flexibility.

## Introduction

SV40, a member of polyomaviradae family, has a small double-stranded circular DNA genome of 5.2 kb. The DNA is complexed with histones, forming a nucleosomal structure similar to cellular chromatin, referred to as a minichromosome. The SV40 structure has been solved at 3.1 Å resolution [1]. The viral capsid, surrounding the viral minichromosome, is a *T* = 7d icosahedral lattice ∼45 nm in diameter. It is composed of three viral-encoded proteins, VP1, VP2, and VP3. VP3 translation initiates from an internal AUG within the VP2 coding sequence utilizing the same translational frame. Thus both proteins share 234 identical amino acids at their carboxy part, and are frequently referred to as VP2/3. VP1 forms the outer shell while VP2 and VP3 bridge between the VP1 shell and the chromatin core. The VP1 monomers are tightly bound in pentamers that are readily formed in the cytoplasm following mRNA translation, through interdigitating β-strands [2]. A single molecule of VP2 or VP3 is tightly attached to each pentamer at its inward facing cavity, through a region close to the C-terminus of VP2/3 [3], [4]. These and other findings indicate that VP1_5_VP2/3 is the building block for SV40 capsid assembly. VP1 has a jelly-roll β-barrel structure [1], [5], with extending N-terminal arm, that carries the DNA-binding domain [6], and ∼60 amino acids long C-terminal arm.

The icosahedral capsid of members of the polyomaviradae family is built of 72 identical VP1 pentamers [7]. 12 of those are surrounded by 5 pentamers each (‘pentavalent pentamers’) and the other 60 by 6 pentamers each (‘hexavalent pentamers’). The puzzle how the variability in contacts between the identical building blocks is achieved has been solved by the elegant X-ray crystal structure study, which has shown that the pentamers are tied together via the long flexible C-arms [5]. Five C-arms extend from each pentamer and insert into the neighboring pentamers in three distinct kinds of interactions. It has been recently shown, by *in vitro* assembly studies, that the correct inter-pentamer bonding in both polyomavirus and SV40 is facilitated by host chaperones [8].

The capsid is stabilized by Ca^++^ and S-S bonds [1]. There are two cation binding sites per VP1 monomer, which appear to stabilize the capsid by “locking” the invading C-arm [1]. Mutations in putative Ca^++^ binding acidic residues suggested that Ca^++^ coordination is important for both virus assembly and for early stages in the infection process [9]. S-S bonds has been observed between monomers of neighboring pentamers, at Cys 104 [1].

Early studies on SV40, performed by a number of groups, revealed that assembly occurred inside the nuclei, and consistently showed that it occurred by addition and organization of the capsomers around the viral minichromosome, rather than by incorporation of DNA into pre-formed capsids (for representative references see [10], [11], [12], [13], [14], [15]). Importantly, empty SV40 particles present in standard virus stocks were demonstrated to be the dissociation products of immature virus rather than an intermediate in viral morphogenesis [13]. Pulse-chase and velocity gradient sedimentation experiments indicated that viral morphogenesis proceeded via a series of intermediates: 100S replicating chromatin→75S intermediate→200S pre-virions→240S mature virions. The 75S nucleoprotein complex was found to rapidly convert to 200S pre-virions, with a full complement of capsid proteins. Assembly intermediates with partial capsids were only seen in studies with virions mutated in VP1 [16], [17]. Notable assembly intermediates also accumulated in virions with mutations at the amino-terminus of the T-antigen gene [18], in a region demonstrated to encode for the co-chaperone J-domain [19], supporting the requirement for chaperones. In addition, rearrangement of nucleosomes around the SV40 genome, driven by the capsid proteins, was shown to be a prerequisite for assembly [20], [21].

All three capsid proteins were found to bind DNA nonspecifically [6], [22] at a high affinity. This raised the question how they recognize the viral minichromosome within the nucleus, in the presence of a large excess of cellular chromatin. Our studies indicated that specific recognition of the SV40 minichromosome is achieved via Sp1 bound at the SV40 encapsidation signal *ses* [23], [24]. Sp1 is a 95 kDa mammalian transcription factor, containing a zinc finger protein motif, by which it binds directly to GC-motifs on the DNA and enhances gene transcription [25]. In SV40 Sp1 binds to the GC-boxes, which are part of the encapsidation signal *ses*, within the regulatory region of the genome.

Our genetic and biochemical studies indicated that Sp1 recruits the capsid proteins to the viral minichromosome, forming an Sp1-VP1_5_VP2/3-*ses* recruitment complex [4], [26]. Furthermore, we found that binding of the VP1_5_VP2/3 complex to *ses* in the presence of Sp1 led to turnoff of both early and late genes [26], thus acting in regulating the transition from replication and transcription to assembly. Sp1 was not found in mature virions [27], indicating that before assembly it was displaced from the viral genome, facilitating nucleosomal reorganization and condensation of the minichromosome [20], [21].

Icosahedral capsid assembly must be an efficient, high fidelity process. The final product is a topologically closed, stable sphere-like particle. Theoretical considerations, supported by experimental results on HBV capsid assembly, suggest that assembly is initiated by a nucleation center, in a slow, rate limiting step [28], [29]. This ensures an extremely robust assembly reaction regardless of protein concentration, avoiding ‘kinetic traps’, which occur when there are too many assembly initiation events and insufficient level of free subunits to allow them to reach completion [30]. In SV40, the Sp1-VP1_5_VP2/3-*ses* recruitment complex (or multiple of those) that binds at a very high affinity to a specific site of the SV40 genome, *ses*, most likely also functions as the nucleation center for assembly. We propose that the 75S species, the only intermediate seen in the early *in vivo* assembly studies, which contains chromatin with a small amount of VP1 and VP2/3 [15], [31], [32], represents the viral genome with the nucleation center attached.

Our recent EM studies, using a VP1 mutant deleted in the C-arm that can bind DNA but cannot assemble, have shown that *in vitro* multiple capsomers bind around single DNA molecules at random locations [27]. We have proposed that this occurs also *in vivo*, after the nucleation center is formed, increasing local capsomer concentration and facilitating rapid, concerted assembly reaction. We have further suggested that these additional capsomers bind at a lower affinity than the nucleation center, allowing their rearrangement around the minichromosome, as required for capsid geometry.

Recombinant VP1 of polyomaviruses contain the information for self-assembly. VP1 of various members of the polyomaviradae family, produced in *E. coli* [33], [34] or in insect cells [35], [36], [37], [38], [39] spontaneously assembles into polymorphic virus-like particles (VLPs) of different sizes. Recently it has been demonstrated that adding chaperones to the assembly reaction leads to a high fidelity (albeit inefficient) assembly of particles of uniform size and shape [8].

We previously reported that SV40 VLPs produced in insect cells were capable of incorporating plasmid DNA in a simple mixing reaction [40]. We showed that intact supercoiled DNA molecules were packaged within the particles, that the particles were infectious and that the transmitted DNA was a biologically active in gene expression and replication. VP2 and VP3 did not enhance their assembly and/or infectivity, suggesting that the VP1 shell is sufficient for these functions. Here we have established an assembly reaction that more closely mimics the *in vivo* process, whereby capsomers assemble around DNA, under physiological conditions, in the presence of as yet unidentified host-cell factors. The results show very high cooperativity of VP1 in the reaction and support the model described above, in which multiple VP1 pentamers first bind to the DNA, which becomes compacted and serves as a scaffold for assembly.

## Results

The experimental system developed here was based on disassembly of VLPs produced in Sf9 cells and their reassembly in presence of supercoiled plasmid DNA. As a VP1 source we used nuclear extracts derived from Sf9 cell infected with recombinant baculovirus expressing VP1. To facilitate functional assay of the assembly process we used a reporter plasmid carrying the *luc* gene. A quantitative assay based on *luc* activity of infected cells, which measures not only particle formation but also their infectivity, has been used throughout the experiments below.

### The disassembly-assembly reaction

SV40 was previously shown to disassemble into VP1 pentamers in the presence of reducing and chelating agents [37], [41], [42]. We have found that the VLPs are significantly less stable than the virus. In contrast to wild type SV40, which required both DTT and EGTA for disassociation, VLPs consisting of VP1, VP2 and VP3 completely dissociated by incubation with either DTT or EGTA alone [37]. For the present study we have chosen to dissociate the particles by DTT and to avoid the use of chelating agents, as they might interfere in subsequent steps of DNA binding and assembly. As observed by EM, the VLPs start dissociating at 5 mM DTT and at 15 mM they are no longer visible (not shown).

As the DNA-binding domain of VP1 is at the internal face of the capsid, we have anticipated that the pentamers would assemble around the DNA. Based on this rationale, the disassembly-assembly reaction has been planned in 3 steps: Step A, dissociation of the VLPs in DTT; step B, the addition of DNA with concomitant dilution of the DTT to allow VP1-DNA binding and assembly; and step C, stabilization of the re-assembled capsids. The complete reaction is described in detail in Materials and Methods.

The requirements for the reaction are summarized in Table 1. The reaction yield, measured as *luc*-transducing units (TU), was highly reproducible, as demonstrated by the small standard error of repeated experiments. As expected, control experiments showed absolute requirement for VP1 and DNA. As an additional control we used a mutant VP1, VP1ΔC, with a deletion of the entire carboxy terminal arm that links between pentamers. As the DNA-binding domain is at the amino terminus of VP1, this mutant retains DNA-binding activity but cannot assemble into capsids [43]. As seen in Table 1, replacement of VP1 by VP1ΔC completely abolished packaging. The requirement for DTT was also absolute, and its presence was required at the dissociation step, A (Table 1). Dilution of the added DTT in the second and third steps allowed the particles to re-assemble around the DNA molecules. The particles were stabilized at pH 5.2.

**Table 1:** Composition of the packaging reaction.

	Composition^a^	Titer×10^3^ TU/ml^b^	Activity (%)
	Complete reaction	1,499±191 (5)	100
	-VP1 control	0 (4)	0
	-DNA control	0 (5)	0
	VP1-ΔC	0 (2)	0
	Purified VLPs	0 (5)	0
**Step A**	-DTT	2±1 (3)	0.1
	-RNase	41±29 (3)	2.7
	-DTT&RNase	8±5 (3)	0.5
**Step B**	-ATP (5 mM)	780±228 (3)	52.0
	-Mg^++^ (5 mM)	784±131 (3)	52.3
	-Glycerol (8%)	983±218 (3)	65.5
	100 mM salt	368±126 (3)	24.5
**Step C**	pH 5.2	1,499±191 (5)	100
	-Ca^++^ (1 mM), pH 5.2	1,519±204 (3)	101
	pH 7.0	556±115 (3)	37.0
	-Ca^++^ (1 mM), pH 7.0	835 (1)	55.7
**Time of DTT addition**	at step A	1,589±431 (5)	100
	at step B	342±211 (5)	21.5
	at step C	25±21 (5)	1.5
	after step C	10±7 (5)	0.6

a Final concentration of the reagents in the reaction mixture at each step is shown in parentheses.

b Average±S.E. The titer is shown as luciferase transduction units. The number in parenthesis represents the number of repeated experiments.

We tested the assembled particles for DNA protection by extensive DNase I digestion, after raising the pH from 5.2 to ∼8, as described in Materials and Methods. The results consistently demonstrated that this treatment did not reduce the yield of *luc*-transducing nanoparticles, indicating that the packaged DNA was DNase resistant.

### The reaction requires cellular factors

**Figure 1:**
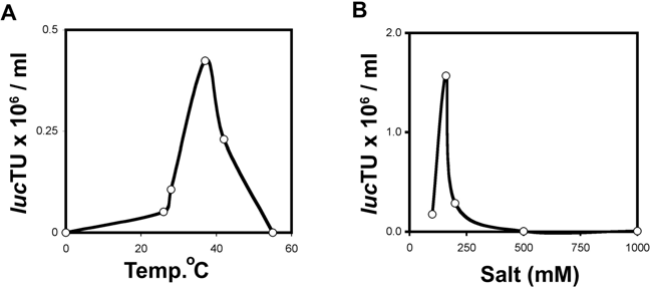
Parameters affecting the in vitro packaging reaction. Effect of temperature (A) and concentration of monovalent salts (B) present during the re-association reaction (step B) on the yield, measured as titer of luc transducing units.

The optimal temperature for the reaction is 37°C and optimal salt concentration was 160 mM monovalent salts (Fig. 1A,B) suggesting an enzymatic activity, presumably provided by the cellular extracts. The optimal salt concentration may also represent a balance between the need, on one hand, to stabilize the particles (VLPs are stabilized at 1 M NaCl [33]), and on the other hand to allow protein-DNA interaction, which are destabilized at a high ionic strength. The requirement for ATP and Mg^++^ (Table 1) is also consistent with the participation of chaperones.

**Figure 2:**
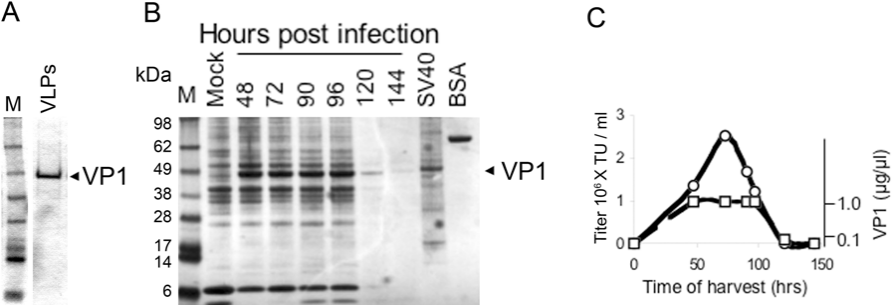
The reaction requires cellular factors. A–VLPs were purified on sucrose gradient from nuclear extracts harvested on day 3. M-molecular weight marker; VLPs–purified VLPs used in the packaging experiment (Table 1). B-Nuclear extracts were harvested from infected Sf9 cells at different time points after infection, as designated on top, and analyzed by SDS-PAGE and Coomassie-blue staining. M-molecular weight marker; Mock-nuclear extract of Sf9 cells infected with wild type baculovirus; SV40-nuclear extract of CV1 cells infected with wild type SV40; BSA-1 µg BSA, a standard for band intensity. C-Activity of the nuclear extracts shown in part B. ○-Packaging activity measured as titer (left ordinate); □-VP1 level estimated from A (right ordinate).

VLPs were purified prior to packaging by sucrose gradient centrifugation from 5 different samples of nuclear extracts (see Fig. 2A for a typical purified preparation). Packaging experiments consistently showed that purification abolished packaging activity (Table 1), indicating that cellular factors were required. Packaging with VLPs isolated from the medium of infected Sf9 cells 5 days post infection, when the cells were lysed, was only ∼1% of the standard reaction with nuclear extracts.

In addition we found that a sharp peak of packaging activity was obtained in nuclear extracts harvested 72 hours post infection (Fig. 2C), while the level of VP1 was constant at 48–96 hours (Fig. 2B,C). This finding suggested that factors in the nuclear extracts other than VP1 participate in assembly.

The nuclear extracts used for the packaging reaction contained significant amounts of Sf9-derived cellular RNA and DNA. RNase treatment following the addition of DTT facilitated removal of free RNA as well as RNA captured within VLPs (data not shown). Consistent with previous findings of others [44], [45], we also found that cellular DNA begins degrading 4 days post infection. At the time of harvest, 3 days post infection, it was high molecular weight, not likely to be packaged within VLPs.

### Stoichiometry of the reaction and cooperativity of VP1

**Figure 3:**
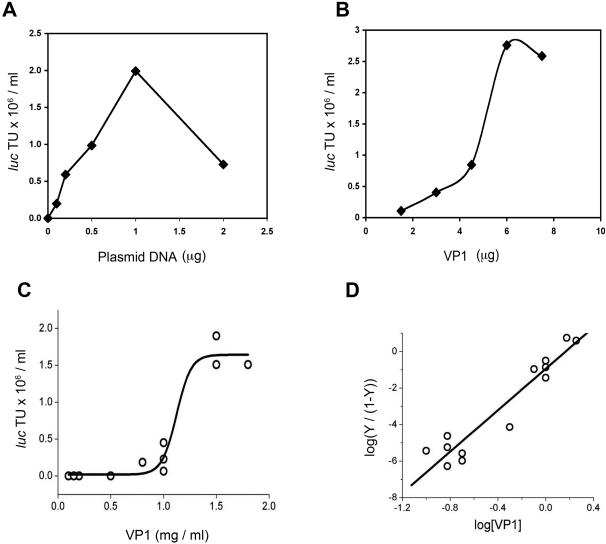
Cooperativity of VP1 in the reaction. Effect of substrate concentration on the reaction yield. A–DNA; B–VP1. The amounts used per reaction, as described in Materials and Methods, is indicated. C-Fourteen different batches of nuclear extracts were assayed for packaging activity. Only 11 distinct data points are seen because of overlap of some of the data points. D-Calculation of Hill coefficient from the same set of data as in C. Y is the fraction of fully assembled VP1, measured as TU.

The reaction yield increased linearly with increasing DNA input, reaching an optimum at 1 µg DNA per reaction (Fig. 3A). Higher DNA concentrations were inhibitory, maybe because they sequestered VP1 pentamers and did not allow the assembly intermediates to go to completion. When VP1 concentration was varied the reaction was sigmoid, indicating cooperativity, and reached saturation at 5 µg VP1 per reaction (Fig. 3B). The optimal VP1:DNA ratio on a weight basis, 5∶1, corresponded to a 1∶1 capsid to DNA molar ratio (MW of a VP1 capsid is ∼15 Mda , and of a 5 Kbp DNA molecule is ∼3 Mda). At high VP1 concentrations the reaction leveled off. It is possible that excess VP1 molecules assembled into empty particles.

Different nuclear extract preparations contained variable levels of VP1. This is not surprising, since VP1 level depends on a number of biological factors that are not fully controlled. Taking advantage of this variability we analyzed 14 different nuclear extract preparations with different VP1 contents. The results (Fig. 3C) showed dramatic sigmoid correlation between reaction yield and VP1 content. Extracts that contained less than 1 µg/µl VP1 had very low activity. Some of the data points were superimposed, therefore only 11 data points are seen in the graph. These data were analyzed according to the Hill equation, which provides a way to quantify cooperativity of a reaction [46]. The resulting Hill coefficient was 5.8±0.6 (Fig. 3D), suggesting that under the present conditions (in presence of nuclear factors) cooperativity was between at least 6–7 partners, presumably VP1 pentamers. For comparison, the Hill coefficient of the cooperative binding of 4 O_2_ molecules to the hemoglobin tetramer is 2.8 [46], [47].

### Characterization of the DNA-containing nanoparticles

**Figure 4:**
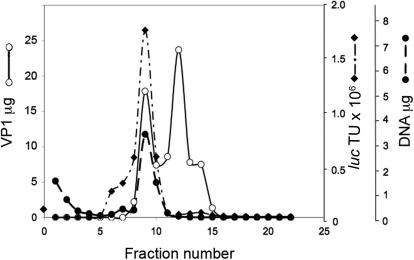
Equilibrium sedimentation in CsCl gradient. The reaction products, 2 ml, were fractionated on a CsCl density gradient in stabilization buffer at pH 5.2 (Materials and Methods). VP1 was analyzed by Coomassie and Bradford, DNA by real-time quantitative PCR and *luc* TU as described in Materials and Methods.

The reaction products were analyzed by equilibrium sedimentation in a CsCl density gradient (Fig. 4). The peak of transducing units coincided with the peak of DNA (analyzed by real-time PCR) at a density of 1.35 g/cm^3^, like wild type SV40. The fraction at the peak contained 3.4 µg DNA, corresponding to 6×10^11^ plasmid molecules or 6×10^11^ packaged nanoparticles. The same fraction contained 1.8×10^6^ transducing units (TU). These data translate to transduction efficiency of 1 per ∼3×10^5^ nanoparticles. For comparison, the infectivity ratio of wild type SV40 is 1∶200 to 1∶500 ([48] and our unpublished results). Thus the nanoparticles are ∼1,000 fold less infective than wild type SV40.

**Figure 5:**
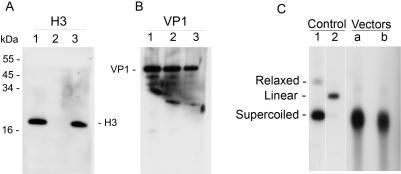
Analysis of the particles. A,B-Proteins were separated by elecrtrophoresis in Tricine buffer on 16% polyacrylamide gel. Western blotting was performed with polyclonal antibody against histone H3 (Upstate) (A) and against VP1 (B). M–size marker; 1–Nuclear extracts used in the packaging reaction; 2–In vitro assembled particles, fraction 9 of Fig. 4. 3–wild type SV40. C-Analysis of the packaged DNA. Particles purified by ultrafiltration were treated with Tris base (200 mM) in presence of 25 mM EGTA and 25 mM DTT for 1 hr at 37°C. DNA was extracted by phenol-chloroform treatment in presence of 1% SDS, and analyzed by Southern blotting with pGL3-control as a probe.

Purified and concentrated by ultrafiltration, the nanoparticles appeared as a single protein (VP1) band on Coomassie-stained (not shown). Western analysis demonstrated that the particles did not contain histone octamers, as detected by antibody against H3 (Fig. 5A, lane 2). Note that H3 histone was detected in the nuclear extract (lane 1) and in wild type SV40 (lane 3). Analysis by VP1 antibody showed that equal amounts of VP1 were loaded on the gel (Fig. 5B). The packaged DNA was released from the purified particles by alkaline treatment and analyzed by electrophoresis. It appeared as a single distinct DNA band (Fig. 5C), suggesting that complete molecules were packaged.

**Figure 6:**
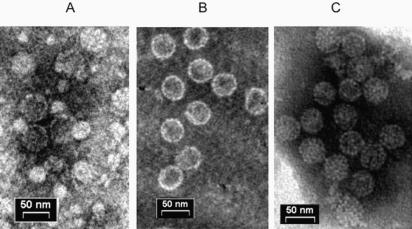
Structure of the nanoparticles. Transmission electron microscopy pictures of (A) VLPs, (B) *in vitro* packaged nanoparticles, and (C) wild type SV40. Samples were adsorbed onto formvar-carbon-coated copper grids and stained with 1% sodium phosphotungstate, pH 7.0. The samples were viewed in a Philips CM-12 electron microscope, using a voltage of 100 kV, and photographed at a magnification of 53,000×.

Under electron microscopy the purified particles appeared well dispersed and of uniform shape and size (∼45 nm, Fig. 6B), like wild type SV40 (Fig. 6C). The purified particles appeared to be more permeable to the stain than wild type SV40, most likely because of the absence of histone proteins, which were shown to occupy much of the space within the particles [49]. It may be noted that prior to packaging many of the VLPs were smaller (Fig. 6A), presumably *T* = 1 and *T* = 3 structures. Thus it appears that the present reaction conditions direct the formation of *T* = 7 particles.

## Discussion

This study demonstrates that capsomers can assemble *in vitro* around DNA, in a reaction that requires host factors. The particles assemble at a stoichiometry of one DNA molecule per capsid, forming uniform particles of the same size, shape and density in CsCl as wild type SV40. Convincing evidence for genuine assembly, rather than non-specific association that protects the DNA from DNase I, is also provided by the finding that VP1-ΔC mutant has not been active in this reaction. This mutant VP1 retains its DNA-binding domain (localized at the N-terminus) and full DNA-binding activity, while it cannot assemble into capsids [43]. Its inability to package argues against non-specific VP1-DNA interaction. True DNA packaging is also suggested by the 1:1 stoichiometry of the reaction and by the supercoiled state of the packaged DNA.

**Figure 7:**
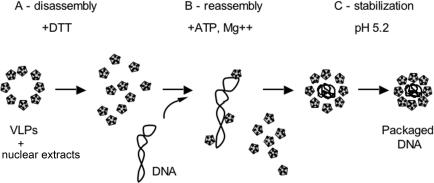
A model for the *in vitro* assembly reaction. The addition of DTT to nuclear extracts (A) leads to disassembly of the VLPs. Following the addition of supercoiled DNA (B) pentamers bind along each DNA molecule. This increases the local concentration of VP1, facilitating concerted assembly. Reassembly may be facilitated by presence of chaperones in the nuclear extracts. Assembly is accompanied by DNA condensation, presumably via the action of topo II. In Step C the capsids are stabilized at pH 5.2.

How does the present assembly reaction proceed? Our data suggest the following scenario (see Fig. 7): The addition of DTT to nuclear extracts leads to disassembly of the VLPs into VP1 pentamers and/or pentamer-clusters (Step A). Following the addition of supercoiled DNA we propose that a number of such units bind, in a rapid reaction, along each DNA molecule (Step B), as shown by our previous EM study with the VP1-ΔC mutant [27]. This increases the local concentration of VP1, facilitating concerted assembly, as seen by the high VP1 cooperativity and Hill coefficient. We predict that DNA-binding is a rapid reaction, and assembly is the rate-limiting step. It is possible that some pentamer-pentamer associations occur prior to DNA-binding, and that pentamer-clusters, rather than single pentamers, serve as building blocks for assembly. However, the possibility that the capsids assemble before they bind DNA is unlikely in view of the absolute requirement for DTT at the beginning of the reaction (Table 1). Thus the DNA appears to function as a scaffold for capsid formation, as described for CCMV [50]. For recombinant VP1 of members of the polyomaviradae family the DNA scaffold is however not obligatory, as we and others have observed that it can assemble into empty capsids in the absence of DNA [33], [34], [35], [36], [37], [38], [39].

Our previous studies have suggested that *in vivo*, as the infection cycle progresses and the level of capsid proteins in the nucleus becomes sufficiently high, they are recruited by Sp1 to *ses*, turning off the early and late promoters and forming the assembly nucleation center [26]. We propose that formation of the nucleation center is followed by binding of multiple capsomers to the minichromosome at a lower affinity. They bind at quasi-random locations, as only part of the DNA is accessible depending on nucleosomal configuration. This binding increases local capsomer concentration, facilitating the highly cooperative assembly reaction as seen here. Following the equilibrium model for virus assembly [28], we suggest that after the initial binding of VP1 pentamers to DNA, interpentameric bonding form and dissociate in a series of equilibrium reactions until correct configuration is achieved and the icosahedral shell is completed. During this process the DNA is compacted, VP1 pentamers rearrange around the molecule as required for capsid geometry and form the proper interpentameric links, out of three different types found in the mature virion [1]. Stability of the icosahedral shell with its low free energy state drives the reaction to completion. In the present reaction the capsids containing DNA are stabilized at a low pH (Fig. 7 Step C).

Could this *in vitro* assembly reaction serve for further studies on *in vivo* assembly? The process described here differs from *in vivo* assembly of SV40 primarily by the absence of VP2 and VP3 and the use of naked plasmid DNA rather than a minichromosome. Nevertheless, the findings obtained from this simplified assembly system are in full agreement with *in vivo* studies. First, the reaction represents assembly around the SV40 genome, rather than insertion of DNA into preformed shells. Second, our data account for the inability of previous investigators to isolate partially assembled intermediates from infected cells, except when mutant VP1 [16], [17], [31], [51] or mutant T-antigen, presumably defective in DnaJ chaperone function [18], [32], were used. Furthermore, the requirement for host factors, for physiological conditions (temperature, pH and salt concentration) and for ATP and Mg^++^ is consistent with the participation of chaperones in this *in vitro* system. A number of investigators have recently reported, based on studies with mutant viruses of both polyoma and SV40, that VP2 and VP3 are not required for the assembly process *in vivo*, but rather for infectivity, egress and for the transport of the viral genome to the nucleus [52], [53], [54], [55]. Consistent with these data the particles produced here are significantly less infective than wild type SV40.

What is the contribution of cellular factors? Recently, the participation of chaperone machinery in assembly *in vitro* of empty particles of polyomavirus and SV40 (in the absence of DNA) has been clearly demonstrated [8]. Specifically those studies suggested that chaperones facilitate the fomration of uniform *T* = 7 nanoparticles, since in their absence polymorphic particles, mostly of smaller size, were formed. Involvement of chaperones in the present assembly reaction is suggested by the temperature and salt profiles of the reaction and the requirements for ATP and Mg^++^. In addition, our recent data (Abd-El-Latif and Oppenheim, unpublished) suggest the participation of topoisomerases, as the reaction yield was affected by the addition of Etoposide , a topo II inhibitor. Topo II most likely functions in DNA compaction, facilitating its packaging inside the particles. Indeed detailed EM studies demonstrated that in the absence of host factors, when purified capsids of polyoma [56] or BK [39] were used, the DNA molecules were attached to the capsids at their external surface rather than being packaged inside.

Additional investigations are needed for full *in vitro* recapitulation of the assembly process, in presence of all three capsid proteins and the viral minichromosome. Nevertheless the present *in vitro* reaction facilitates an insight into the assembly reaction, and will serve for further biochemical and biophysical studies using both wild type and mutant VP1, and as a basis for identification of participating host factors.

Finally, the present reaction can be utilized to produce DNA-containing nanoparticles as a safe gene therapy vector. As the DNA used here is propagated in bacteria, there is no need for the SV40 *ori*. The absence of competing cellular chromatin relieves the requirement for the packaging signal *ses* and for Sp1. Thus plasmids without any SV40 sequences may be packaged, providing the vector with additional safety margin [57]. Furthermore, the absence of histones provides space and facilitates packaging of significantly larger plasmids (plasmids as large as 17 kb were shown to be packaged [57]), another significant advantage for gene therapy vectors. Together with the development of procedures for purification and concentration, we have demonstrated efficient gene delivery of the *luc* reporter gene, constructed as described here, to the liver in mice (Fig. 1C in ref. [58]). Importantly for gene therapy, we and others have found that SV40 vectors do not elicit cellular immune response [58], [59]. Potential applications and merits of *in vitro* constructed SV40-based vectors for gene therapy have been recently discussed [60], [61].

## Materials and Methods

### Cell cultures and media

*Spodoptera frugiperda* (Sf9) cells were grown at 27°C in serum-free Bio-insect medium containing glutamine, penicillin, streptomycin and amphotericin (Biological Industries, Israel). COS-1 cell-line is a derivative of CV1 (ATCC # CCL70) harboring the SV40 T-antigen gene [62]. COS-1 cells were cultured in high glucose Dulbecco's modified Eagle's medium containing glutamine, penicillin, streptomycin, and 10% FBS.

### Production of recombinant VP1 and DNA

Recombinant baculovirus expressing VP1 (Swiss-Prot P03087, PDB 1SVA) from the polyhedrin promoter [37] were propagated in Sf9 cells. High titer virus stocks (>10^9^ pfu/ml) were used to infect logarithmic cultures of Sf9 cells, at moi 10, for VP1 production. Seventy two hours post infection the cells were harvested by centrifugation and nuclear extracts were prepared by a procedures adapted from Schreiber et al. [63]. Nuclear extracts were stored in aliquots at −80°C. The VP1ΔC mutant is deleted for the complete 65 amino acid C-arm and carries a His tag at its carboxy end [27].

Plasmid pGL3-control (Promega cat # E1611), which carries the *luc* reporter gene expressed from the early SV40 promoter, was used for the *in vitro* packaging experiments. It was prepared in *E. coli* and purified using Qiagene EndoFree Plasmid Purification Giga kits.

### Protein analysis

Total protein was determined by the Bradford assay. The amount of VP1 in nuclear extracts was estimated from Coomassie-stained SDS-PAGE by comparing band intensities with known amounts of BSA.

### *In vitro* assembly reaction

Assembly reactions were conducted in 3 steps. A typical reaction was carried in microtubes as follows: In step A, 5 µl Sf9 nuclear extract, containing ∼5 µg of VP1 were treated with DTT to a final concentration of 15 mM, and with 10 µg RNase, in a final volume of 10 µl at 37°C for 20 minutes. In step B the DNA mix (containing 1 µg pGL3-control DNA, 10 mM ATP, 20 mM Hepes-KOH buffer at pH 7.9, 80 mM KCl, 40 mM NH_4_Cl, 10 mM MgCl_2_, 16% Glycerol and 0.08% NP-40, in a final volume of 10 µl) was added to the treated nuclear extracts (step A), and the reaction was further incubated at 37°C for 1 hour. Note that at step B the DTT was diluted 2 fold and salt concentration brought to 160 mM. For step C the packaging reaction was kept on ice overnight after the addition of 10 µl stabilization buffer (150 mM sodium acetate buffer pH 5.2, 3 mM CaCl_2_ (final concentration 1 mM), 120 mM KCl and 40 mM NH_4_Cl, keeping the salt concentration at 160 mM), bringing the total volume to 30 µl. The mixture was treated with 0.5 unit of DNase I (Promega) on ice for 10 minutes. The reaction was stopped by adding 10 µl of chloroform, the mixture was vortexed, separated by centrifugation and the assembled particles were recovered in the aqueous layer. The reaction was upscaled 1,000 fold in 50 ml tubes without loss of activity.

### Quantification of the reaction yield

The reaction yield was measured by titrating infectivity of the nanoparticles as *luc*–transducing units (TU) in COS-1 cells, using an end-point dilution assay [58]. COS-1 cells were infected by serial dilutions of the reaction mixture, LUC activity was measured 2 days later, the data plotted and TU titer was determined by extrapolation to null LUC activity. Titers obtained by this method were consistent with titers measured as infective centers, scored by *in situ* hybridization of infected CMT4 monolayers [24].

### DNase resistance of the packaged DNA

The reaction mixture was routinely treated with DNase I before measuring the titer yield. However during the course of this study the pH of the stabilization step, just prior to DNase treatment, was changed from 7.0 to 5.2, which is not favorable for DNase I activity. Therefore DNase resistance of the packaged DNA was re-tested after the pH was raised to ∼8 with 1 M Tris base, and Mg^++^ was added to a final concentration of 10 mM. DNase I resistance was tested by the addition of 0.5 units per reaction (containing 1 µg pGL3-control DNA) and incubation for 30 min at 25°C. Reconstruction experiments demonstrated that 1 µg of plasmid DNA added to a mock reaction mixture was extensively digested under these conditions by 20 min.

### Equilibrium sedimentation in a CsCl gradient

2 ml of the reaction products were centrifuged in CsCl in stabilization buffer (ρ = 1.34 g/cm^3^) at 40,000 rpm, 4°C for 40 hrs. Fractions were collected from the bottom, refractive index was measured and the fractions were dialyzed against stabilization buffer. Quantitative real-time PCR was performed using SYBR green in a Light Cycler machine (Roche), with primers flanking the SV40 polyA signal generating a 177 bp PCR fragment (SV40 coordinates 2533-2710): 5′-ACATTGATGAGTTTGGACAAACCAC-3′ and 5′-CCCCTGAACCTGAAACATAAAATG-3′.

### Purification and concentration of the nanoparticals

For some experiments the particles were purified and concentrated ∼30 fold by stirred-ultrafiltration under Argon using XM300 membrane (Millipore). This step was performed following DNase I and Chloroform treatment. The concentrate was resuspended in PBS (half the original volume) and re-filtered 3 additional times. In later experiments we found that using saline instead of PBS substantially enhanced viability of the assembled particles (measured as infectivity) during prolonged storage at −20°C.
